# Practical microalgal supplementation: reducing ammonia emission from manure in commercial layer production

**DOI:** 10.1186/s40104-025-01264-z

**Published:** 2025-10-27

**Authors:** Zihao Yu, Xin Ma, Tiao Long, Haiyang Li, Shiyin Xie, Yiheng Deng, Weikang Deng, Xindi Liao, Sicheng Xing, Jingyuan Chen

**Affiliations:** 1 College of Animal Science, South China Agricultural University, Guangzhou, 510642 Guangdong China; 2 Integrative Microbiology Research Centre, South China Agricultural University, Guangzhou, 510642 China; 3 Guangdong Provincial Key Lab of Agro-Animal Genomics and Molecular Breeding, and Key Laboratory of Chicken Genetics, Breeding and Reproduction, Ministry Agriculture, Guangzhou, 510642 Guangdong China; 4 National-Local Joint Engineering Research Center for Livestock Breeding, Guangzhou, 510642 Guangdong China

**Keywords:** Ammonia, Flavor omics, Hen manure, Microalgal, Static odor production

## Abstract

**Background:**

The rapid development of intensive layer breeding has intensified odor pollution that must be paid attention to for the green transformation of the industry. This study used Jingfen No.6 laying hens as the model to systematically evaluate the regulatory effect of compound microalgal powder (*Chlorella vulgaris*:*Spirulina platensis*:*Haematococcus pluvialis* = 3:1:1, 1:3:1, 1:1:3) on ammonia (NH_3_) emissions from laying hen manure.

**Results:**

Through analysis of the static NH_3_ production in manure, it was found that the NH_3_ emissions within 24 h in the experimental group with 0.50% compound microalgal powder added were reduced to 6.27–16.84 mg (vs. control: 28.29 mg), achieving a 40.47%–77.84% reduction. GC/MS and 16S rRNA sequencing analyses indicated that the compound microalgal powder intervened in the remodeling of the microbial community and nitrogen metabolism network in manure, driving the transformation from inorganic nitrogen to organic nitrogen, mitigated the proliferation of NH_3_-producing bacteria (such as *Escherichia coli*, *Klebsiella pneumoniae*, *Kurthia*, and *Proteus*), and increased the abundance of acid-producing bacteria (such as Leuconostocaceae and Lactobacillaceae). The *Spirulina platensis* powder group had the best emission reduction effect (reduced by 77.84%), and its mechanism was closely related to the mitigation of Gram-negative bacteria activity by phycocyanin and increased synthesis of aromatic compounds, such as 2,3,5-trimethyl-6-ethylpyrazine.

**Conclusions:**

This study revealed the mechanism by which the compound microalgal powder reduces NH_3_ emissions by regulating the proliferation of acid-producing bacteria, reshaping the nitrogen metabolism network, and mitigating the activity of NH_3_-producing bacteria, while providing theoretical and data support for the development of environmentally friendly feed.

**Graphical Abstract:**

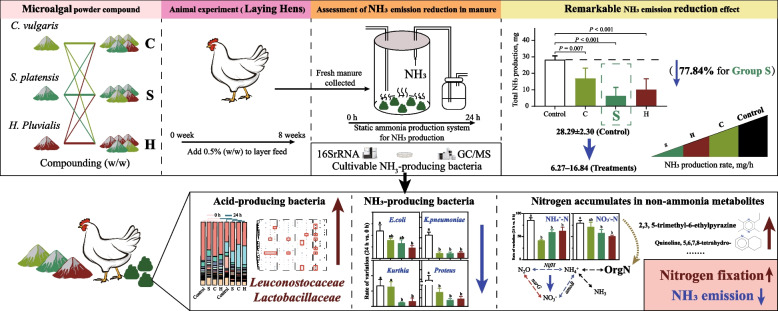

**Supplementary Information:**

The online version contains supplementary material available at 10.1186/s40104-025-01264-z.

## Highlights

- Dietary supplemental microalgal powder reduces NH_3_ emissions from laying hen manure.
- Nitrogen deposition and transformation in manure regulate NH_3_ production.
- Mitigation of NH_3_-producing bacterial proliferation was the primary mechanism.
- *Kurthia *and *Proteus* were identified as principal NH_3_-producing bacteria.
- Lactobacillaceae and Leuconostocaceae mitigates the NH_3 _emissions.

**Supplementary Information:**

The online version contains supplementary material available at 10.1186/s40104-025-01264-z.

## Introduction

The rapid development of intensive layer breeding has made the odor pollution of poultry houses a key bottleneck restricting the green transformation of the industry [1]. Notably, the health hazards posed by malodorous gases to livestock exhibit a pronounced dose- and time-dependent relationship, with prolonged exposure to threshold ammonia (NH_3_) environments inducing systemic harm to poultry [2–4]. As the primary odor component of poultry houses, NH_3_ not only directly irritate poultry eyes and reduces their feed intake, but also weakens immune defences during low concentration chronic exposure, leading to frequent respiratory diseases and compromising respiratory health [5–7]. Ammonia emissions from hen operations constitute the predominant source within China’s livestock sector, accounting for 29.0% of its emissions [8]. These emissions contribute 14.5% of China’s total ammonia emissions, given that agricultural activities represent 88.0% of the national total, with livestock farming comprising 56.7% of agricultural sources [9]. Emitted NH_3_ undergoes complex atmospheric transformations: it is readily converted to ammonium nitrogen and combined with particulates to form PM2.5 (such as secondary aerosols), intensifying the greenhouse effect by combining with the ozone layer and forming acidic compounds through nitrogen deposition, leading to acid rain phenomenon, eventually causing chain ecological risks such as soil acidification and water pollution [10, 11].

The NH_3_ emission in laying hens is mainly composed of three interfaces: blood ammonia metabolism, intestinal microbial nitrogen transformation and manure ammonia degradation. The production of NH_3_ mainly occurs in the manure management stage [12–14]. At present, most intensive layer farms adopt mechanized track manure cleaning technology, which leads to the manure that has not been cleaned in time often staying in the house for up to 12–48 h [15]. Nitrogen-containing organic matter in feces is gradually decomposed into uric acid, urea, and ammonium nitrogen under the action of urease, and then converted into volatile NH_3_ under alkaline conditions [16, 17]. This process is regulated by the structure of gut microbiota, the physicochemical properties of manure, and environmental parameters [18, 19]. It is worth noting that the production of NH_3_ is the inevitable result of protein decomposition, and the essence of its production is the process of nitrogen mineralization, in which microorganisms play an important and key role. Dominant NH_3_-producing bacteria catabolize nitrogen-containing substrates to obtain nitrogen sources for further synthesis of microbial proteins, and in this process, NH_3_ is produced as a by-product of the nitrogen cycle [20, 21]. Common NH_3_-producing bacteria are mainly Gram-negative bacteria, such as *Escherichia*, *Klebsiella*, *Proteus* and *Pseudomonas* [22, 23].

The current NH_3_ emission reduction technology focuses on two directions: nutritional optimization and environmental control. The application of dietary protein precision and fermented feed has a certain effect on NH_3_ reduction [24, 25]. Reducing crude protein level without affecting poultry performance can reduce NH_3_ emission [26]. Replacing soybean meal with fermented cottonseed meal also has a certain potential to reduce NH_3_ emission [27], but such nutritional strategies face problems such as amino acid balance and palatability limitations. In contrast, microalgal additives exhibit multi-pathway regulatory advantages due to their unique bioactive components and nitrogen metabolism regulation functions [28, 29]. Microalgal additives are rich in proteins, vitamins, minerals and antioxidant substances [30], and have shown great potential in enhancing poultry immunity, regulating intestinal microecology and improving nitrogen metabolism [31]. The cell wall polysaccharides of microalgae can immobilize ammonium ions through molecular adsorption [32, 33]. Their rich phenolic substances can mitigate urease activity [34], and their high content of chlorophyll derivatives can directly participate in the electron transport chain of the nitrogen cycle [35]. The differences in microalgae varieties endow them with different functions. For instance, *Chlorella vulgaris* is rich in proteins, polysaccharides, and chlorophyll, and can regulate the intestinal microbial community to lower the pH value of the manure of laying hens, thereby mitigating urease activity and reducing ammonia emissions [36]. *Spirulina platensis* contains abundant phycocyanin, chlorophyll, and polyphenolic compounds, and exhibits significant antioxidant and antibacterial effects, promoting the growth of acid-producing beneficial microorganisms and reducing the pH value of the manure of laying hens, thereby decreasing ammonia production [37]. *Haematococcus pluvialis* is rich in astaxanthin and can enhance the host's antioxidant and immune capabilities, regulate the microbial community structure, mitigate the proliferation of harmful bacteria in chicken manure, and reduce ammonia emissions [38]. The functions of single algal powder are relatively limited. However, a composite algal powder additive formulated from multiple algal powders may compensate for the functionalities of various microalgae. *Chlorella vulgaris* and *Spirulina platensis* mitigate urease activity and directly reduce ammonia emissions by regulating the intestinal environment; *Haematococcus pluvialis* reduces ammonia production indirectly by improving the enteric antioxidant and immune capabilities. Combination of these three algal species may exert a synergistic effect, reducing ammonia emissions through multiple pathways. Currently, in the agricultural field, research and application of composite additives are advancing towards efficiency, environmental-friendliness, and multi-functionality [39–41]. However, research on composite microalgae additives is limited, particularly regarding its impact on laying hen production. Feeding microalgal additives may achieve the whole chain regulation of microorganisms through the “gut-manure” pathway. Feeding microalgae to laying hens directly affects the microbial composition of laying hen manure by affecting the intestinal microbial colonies of laying hens. The changes in microbial structure of laying hen manure are necessary to mediate the law of NH_3_ production. However, the research exploring the potential of microalgal additives for reducing NH_3_ emissions in laying hens remains relatively limited.

In order to systematically evaluate the NH_3_ reduction efficiency of microalgal additives, Jingfen No.6 laying hens were used as the model in this study. Common microalgal additives (*Chlorella vulgaris*, *Spirulina platensis* and *Haematococcus pluvialis*) were mixed in different proportions to prepare composite microalgal powder and fed to the laying hens at 0.50% addition ratio. The key nitrogen metabolites were identified by flavor omics, and the key microorganisms were identified by amplicon sequencing and culturable bacteria isolation to reveal the “microbial-metabolism-NH_3_” interaction network. Our study has established a multi-algal synergistic model, and is attempting to identify the optimal composite algae-flour additive formulation to provide theoretical data for the development of eco-friendly feed. The results of this study will help achieve the goal of nitrogen emission reduction in animal husbandry and provide methodology for pollution control of livestock and poultry breeding.

## Materials and methods

### Feeding experiment and manure collection

According to previous studies [42], the microalgae species commonly used as microalgal powder additives in the Chinese market include *Chlorella vulgaris *(*C. vulgaris*), *Spirulina platensis *(*S. platensis*), and *Haematococcus pluvialis *(*H. pluvialis*). In this study, these three microalgal additives were blended at different ratios (w/w) to prepare three compound microalgal powders: *C. vulgaris*:*S. platensis*:*H. pluvialis* = 3:1:1 (Group C), 1:3:1 (Group S), and 1:1:3 (Group H) [43–45]. The compound microalgal powders were supplemented in laying hen diets at 0.50% (w/w) and fed to 500-day-old Jingfen No.6 laying hens (a Chinese indigenous breed currently under commercial promotion [46]) for 8 weeks. A blank control group (Group Control, without microalgal supplementation) was included. Each treatment consisted of 6 replicates with 24 laying hens per replicate. Hens were housed in 6 cages per replicate (cage dimensions: 45 cm × 60 cm × 42 cm), distributed equally across upper, middle, and lower tiers (2 cages per tier). Stocking density was maintained at 4 hens per cage. Prior to experimental commencement, body weights were standardized across groups to minimize inter-individual variation. All laying hens were maintained in an automated layer facility under controlled environmental conditions (20–25 °C; 55%–65% relative humidity; 16-h photoperiod (05:00–21:00)) with ad libitum access to feed and water. Microalgae powder (nutrient composition detailed in Supplementary Table S1) was supplemented to basal diets (composition in Supplementary Table S2).

### Static NH_3_ production experiment

**Fig. 1:**
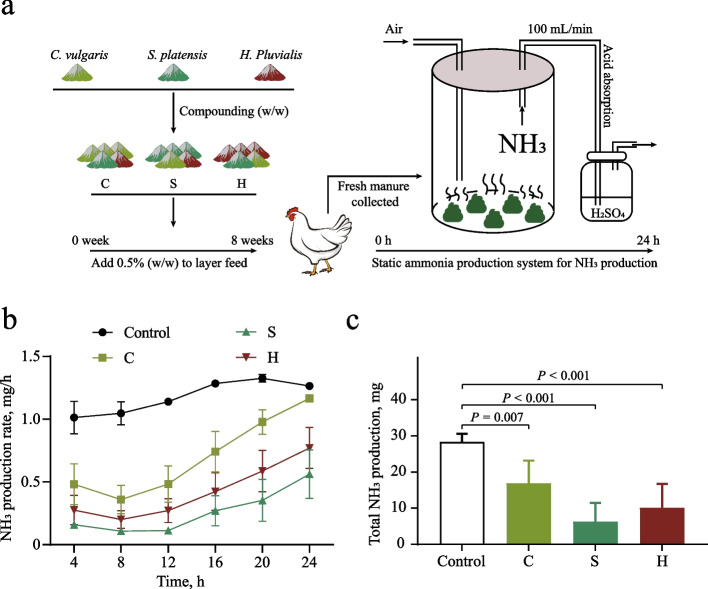
NH_3_ emission data from treatment groups. **a** Schematic of the experimental design. Manure from hens fed compound microalgal powder was placed in a static NH_3_ production system for 24 h NH_3_ detection. Formulations: *Chlorella vulgaris*:*Spirulina platensis*:*Haematococcus pluvialis* = 3:1:1 (Group C), 1:3:1 (Group S), and 1:1:3 (Group H); the control group received a normal diet. **b** The generation pattern of NH_3_ within 24 h. **c** Total 24-h NH_3_ emissions. Significant differences determined by Tukey’s test (*P* < 0.05)

After the feeding experiment, fresh hen manure was collected and placed into a static NH_3_ production system. Through the principle of negative pressure suction, the NH_3_ naturally produced by the manure was absorbed by sulfuric acid absorption solution (0.01 mol/L). The absorption solution was replaced every 4 h, and the NH_3_ concentration was measured to calculate the total absorbed NH_3_. The static NH_3_ production test lasted 24 h, with the airflow rate maintained at 100 mL/min. A schematic of the system is provided in Fig. 1a. Manure samples from each treatment group were collected at 0 and 24 h. A subset of samples was flash-frozen in liquid nitrogen and stored at −80 °C for physicochemical and biological analyses. The remaining samples were mixed with 50% glycerol (v/v) and stored at −80 °C for subsequent culturable bacterial isolation and identification.

### Determination of physicochemical properties

The NH_3_ concentration in the sulfuric acid absorption solution was determined by the Nessler reagent method, and the total NH_3_ production was calculated based on the measured concentration. Ammonium nitrogen and nitrate nitrogen contents in the manure samples were quantified using commercial assay kits (Soil Ammonium Nitrogen Content Assay Kit, Solarbio, UK; Soil Nitrate Nitrogen Content Assay Kit, Solarbio, UK), following the manufacturer’s protocols.

### Metabolite quantification based on flavor omics

The hen manure samples (*n* = 48) were analyzed by gas chromatography-mass spectrometry (GC-MS). Metabolite extraction: A single headspace sampling bottle was used separately, and 10 μL (50 mg/L) of n-alkanes mixed standard was added to each bottle. GC-MS analysis: In the SPME cycle of the PAL rail system, the incubate ion temperature was 60 °C; preheating time was 15 min; incubation time was 30 min; and desorption time was 4 min. The GC-MS analysis was performed using an Agilent 7890B gas chromatograph interfaced with a 5977B mass spectrometer. The system utilized a DB-Wax. Samples were injected in Splitless Mode. Helium was used as the carrier gas; the front inlet purge flow was 3 mL/min; and the gas flow rate through the column was 1 mL/min. The initial temperature was kept at 40 °C for 4 min; then raised to 245 °C at a rate of 5 °C/min; and maintained for 5 min. The injection, transfer line, ion source, and quad temperatures were 250, 250, 230, and 150 °C, respectively. The energy was −70 eV in electron impact mode. The mass spectrometry data were acquired in scan mode with an *m*/*z* range of 20–400 and solvent delay of 2.37 min. Raw data processing (including peak extraction, baseline filtering/calibration, peak alignment, deconvolution analysis, peak identification, integration, and spectral matching of peak areas) was conducted with LECO ChromaTOF 4.3X software and the NIST 20 mass spectral database.

### Amplicon sequencing and quantitative PCR analysis

The hen manure genomic DNA (*n* = 48) was extracted using the E.Z.N.A.^®^ Soil DNA Kit (Omega Bio-tek). The extracted DNA was stored at −80 °C until amplicon sequencing and quantitative polymerase chain reaction (qPCR) analysis. Firstly, the extracted DNA samples underwent bacterial 16S rRNA gene sequencing analysis, targeting the V4–V5 region with specific barcoded primers 515F/926R. After the library was successfully constructed and quality control, sequencing was performed on an Illumina MiSeq/Novaseq platform to obtain paired-end reads. The bioinformatics process included quality control of paired-end reads, denoising and other optimization strategies to generate amplicon sequence variants (ASVs). Subsequent analyses were conducted based on ASVs representative sequence and quantified abundance data. Secondly, the extracted DNA samples were used for qPCR quantification of 4 NH_3_-producing bacteria (*Escherichia coli*, *Klebsiella pneumoniae*, *Kurthia*, and *Proteus*)*.* The selection of these bacteria was based on culturable bacterial isolation and identification results. Total bacterial abundance was quantified using 16S rRNA gene amplification. Specific primer information was shown in Supplementary Table S3. Quantification was performed by qPCR using a Bio-Rad CFX96 system (Bio-Rad, USA).

### Expression quantification of nitrogen cycle-related genes

Expression of nitrogen cycle-related genes in hen manure samples was quantified, we analyzed 8 nitrogen cycling-related genes (*amoA*, *amoB*, *narG*, *nirS*, *nirK*, *norB*, *nosZ*, and *NifH*). Primer sequences are provided in Supplementary Table S4. Total RNA was extracted from hen manure (*n* = 48) using the Quick-RNA™ Fecal/Soil Microbe MicroPrep Kit (ZYMO, USA). Subsequently, transcripts of nitrogen cycling-related genes (*n* = 8) were quantified by quantitative reverse transcription polymerase chain reaction (qRT-PCR) using the Bio-Rad CFX96 system. Relative expression was calculated using the 2^−ΔΔCt^ method.

### Isolation and identification of NH_3_-producing bacteria

The hen manure samples were homogenized in sterile water at a 1:9 (w/w) ratio and vortexed for 20 min. The suspension was serially diluted in sterile phosphate-buffered saline (pH = 6.8) and spread onto Urea Agar Base agar plates supplemented with 2% urea. Plates were incubated at 37 °C until red colonies appeared. Individual colonies were purified and subcultured for further analysis. Bacterial identification was performed by PCR with universal primers 27 F and 1492R. Qualified PCR products were subjected to Sanger sequencing (Sangon Biotech, Shanghai, China). The obtained 16S rRNA gene sequences were aligned using MAFFT v7.0, filtered with TrimAl v1.4 to remove low-quality regions, and trimmed to retain conserved regions. A maximum-likelihood phylogenetic tree was constructed with FastTree v2.1 and visualized with tvBOT.

### Statistical analysis

Statistical analyses were performed using SPSS 22.0 (IBM Corp, USA) and Prism 8.0.1 (GraphPad Inc., USA). Mean separation among treatments were determined by one-way ANOVA. Spearman's correlation coefficients between normalized bacterial abundances and target metabolites were computed in R 4.3.0. Network plots illustrating the abundance relationships between target metabolites and bacterial communities were generated with Gephi 0.9.2 software. The heatmap was created in R 4.3.0 software.

## Results

### Compound microalgal powder supplementation reduces NH_3_ emissions

To evaluate the potential of compound microalgal powder to reduce NH_3_ emissions from laying hen manure, we collected manure and quantified total 24-h NH_3_ emissions using a static system (Fig. 1a). Following supplementation with compound microalgal powder, treatment groups showed significantly lower per-unit-time NH_3_ emissions than the control group (*P* < 0.05). Cumulative 24-h emissions were 6.27–16.84 mg in experimental groups versus 28.29 ± 2.30 mg in controls, representing a 40.47%–77.84% reduction. Despite a temporal increase in emissions across all groups (Fig. 1b), the 24-h cumulative NH_3_ remained significantly lower in treatment groups (Fig. 1c), demonstrating the efficacy of microalgal powder in reducing manure NH_3_ emissions.

### Microalgal powder alters inorganic nitrogen dynamics and nitrogen cycling gene expression

**Fig. 2:**
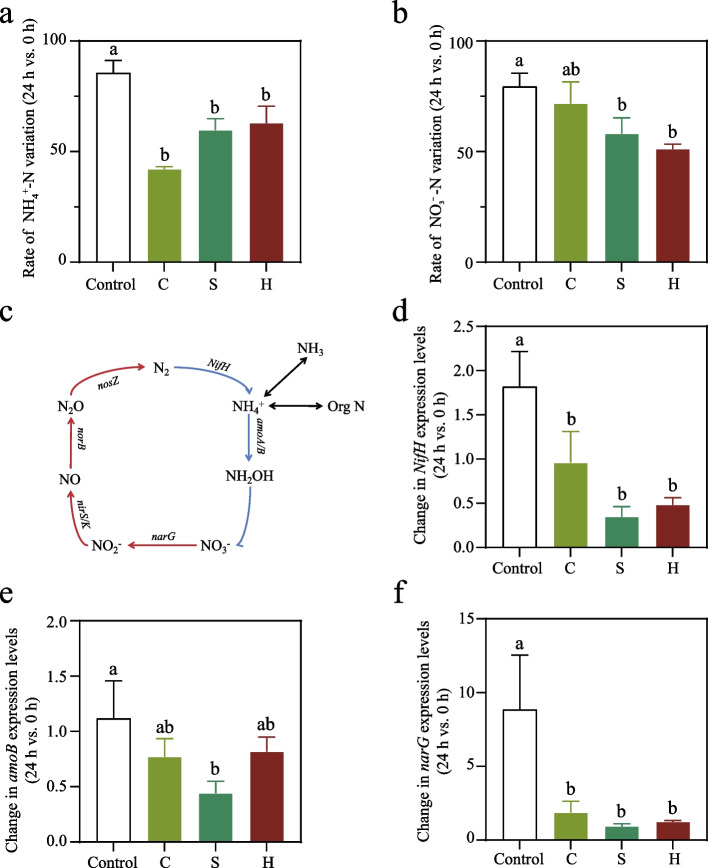
Inorganic nitrogen dynamics and nitrogen-cycling gene expression. **a** The rate of NH_4_^+^-N variation (%) between 0 and 24 h. **b** The rate of NO_3_^−^-N variation (%) between 0 and 24 h. **c** Schematic of nitrogen flow. **d**–**f** Change in *NifH*, *amoB*, *and narG* expression levels between 0 and 24 h. Values are presented as mean ± SD. Different lowercase letters indicate significant differences among groups (*P* < 0.05, Tukey's test)

To elucidate the mechanism behind reduced NH_3_ emissions, we analyzed inorganic nitrogen changes in manure. The rate of change (24 h/0 h) in ammonium nitrogen (NH_4_^+^-N) content decreased significantly in all treatment groups (Fig. 2a), with similar reductions in nitrate nitrogen (NO_3_^−^-N) change rates (*P* < 0.05; Fig. 2b). This indicates greater inorganic nitrogen consumption in treatment groups without proportional NH_3_ production. We further examined expression of nitrogen-cycling genes (Fig. 2c and Supplementary Fig. S1), such as *amoA*, *amoB*, *narG*, *nirS*, *nirK*, *norB*, *nosZ*, and *NifH*, hoping to explain the whereabouts of nitrogen from the perspective of nitrous oxide (N_2_O) emissions. Change rates (relative to d 0) for *NifH*, *amoB* and *narG* are shown (Fig. 2d–f). The expression level of *NifH* increased in control group, while it showed a decreasing trend in the experimental group (Fig. 2d). The expression level of *amoB* in the control group changed little, but a decreasing trend was shown in all treatment groups (Fig. 2e). While the expression level of *narG* was upregulated across all treatment groups, but the upregulation ratio in the experimental group was significantly lower than that in control group (*P* < 0.05; Fig. 2d). These results suggest that compound algal powder increases the consumption of inorganic nitrogen without enhancing nitrogen flux toward N_2_O.

### GC-MS revealed the changes of key nitrogen-containing metabolites

Based on the above results, we speculate that feeding laying hens with compound microalgal powder causes the inorganic nitrogen in their manure to deposit as organic nitrogen during the static process, resulting in a reduction in NH_3_ emissions (Fig. 2c).

**Fig. 3:**
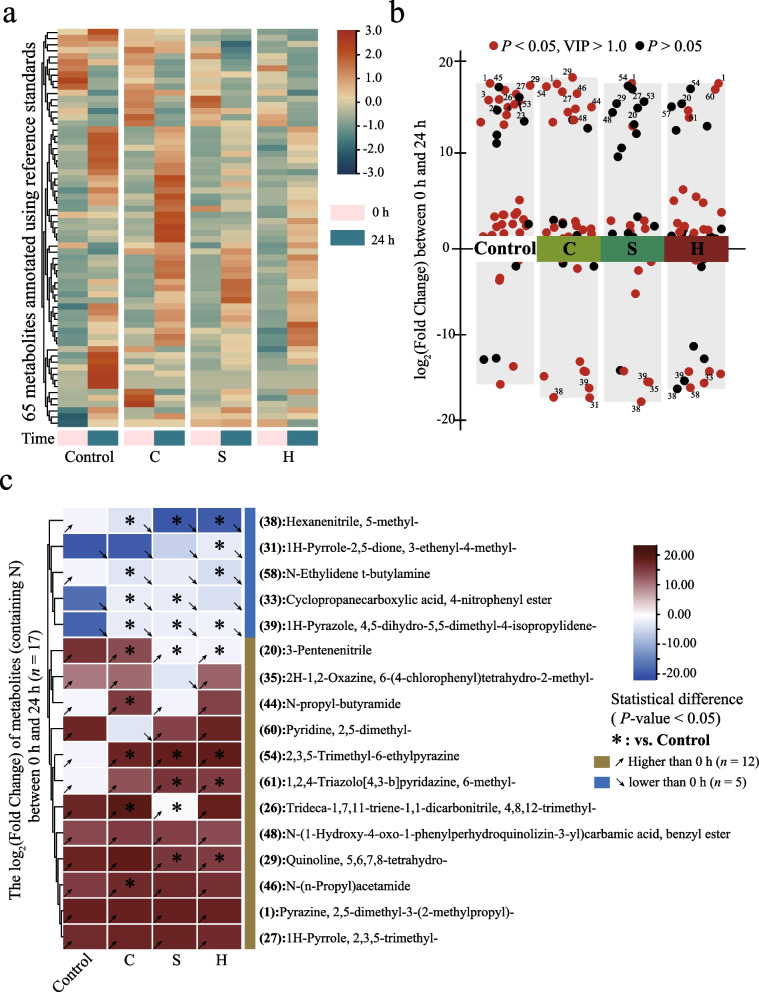
Nitrogen-containing metabolites in manure based on GC/MS. **a** The 65 nitrogen-containing metabolite landscapes obtained. **b** Volcano map depicts the types of different metabolites in each treatment group. A red bubble means *P* < 0.05 and VIP > 1.0. **c** Distribution of change rates of 17 key differential metabolites. An asterisk means *P* < 0.05

Based on GC/MS, we conducted flavor omics analysis on the manure of laying hens and identified 65 nitrogen-containing metabolites (Fig. 3a). Based on the differences in nitrogen-containing metabolites in laying hen feces before and after static NH_3_ production, we marked the metabolites (Top 10) with significant changes in content in each treatment group (Fig. 3b), and presented the content change rates of 17 metabolites with relatively high abundances among them in the form of a heat map (Fig. 3c). Among them, we defined 12 metabolites as synthetic metabolites and 5 metabolites as consumable metabolites. The results of the heat map indicated that the change rates of most key nitrogen-containing metabolites (12/17) showed significant differences among the treatment groups (*P* < 0.05). After the intervention of compound microalgal powder, among the 5 consumptive metabolites in manure, the consumption of 3 metabolites decreased (No.31, 33 and 39), the consumption of 2 metabolites increased (No.38 and 58), and among the other 12 synthetic metabolites, the synthesis of 4 metabolites increased (No. 44, 46, 54 and 61) and that of 1 metabolites decreased (No. 29).The synthesis of metabolite No. 26 (Trideca-1,7,11-triene-1,1-dicarbonitrile, 4,8,12-trimethyl-) increased in group C and decreased in group S (*P* < 0.05). The differences in the change rates of these nitrogen-containing metabolites among the treatment groups may be the main cause of NH_3_ emissions.

### Metabolites related to NH_3_ emission reduction mediated by microalgal powder

**Fig. 4:**
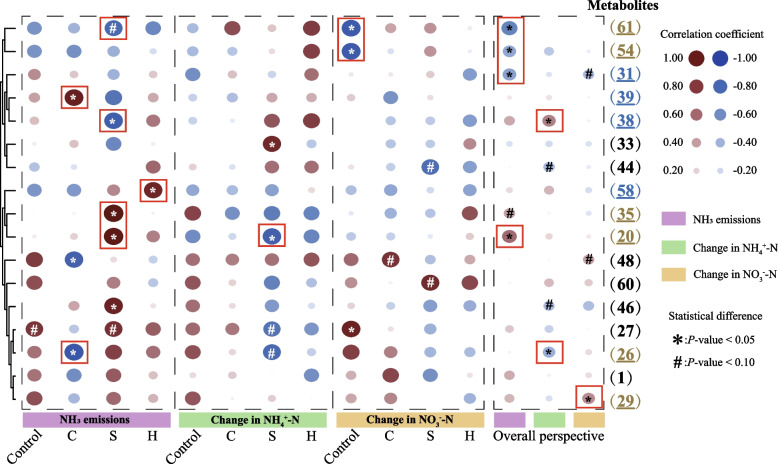
Correlation between the key metabolites, NH_3_ emissions, and inorganic nitrogen. An asterisk means *P* < 0.05 and the “#” indicates *P* < 0.10

We attempted to conduct a correlation analysis between the variation amounts of these 17 metabolites and the variation amounts of ammonia emissions and inorganic nitrogen, and screen out the types of metabolites closely related to the flow of nitrogen elements (Fig. 4).

The analysis results of the bubble heat map show that the changes of nitrogen-containing metabolites are closely related to the NH_3_ emissions. From the perspective of inorganic nitrogen, although only metabolite No. 29 was found to have a significant positive correlation with the change in NO_3_^−^-N at the overall level (*P* < 0.05), in the control group, we found more key metabolites, such as metabolites No. 27, No. 54 and No. 61, and all the above metabolites were synthetic metabolites. Combined with the analysis of Fig. 3c results, under the intervention of compound microalgal powder, the accumulation of the synthesis of metabolites 54 and 61 and the reduction of the synthesis of metabolite 29 were the reasons for the decrease in NO_3_^−^-N content and the reduction in NH_3_ emissions. In other words, the intervention of compound microalgal powder may cause NO_3_^−^-N and metabolite 29 to flow towards metabolites 54 and 61.

The correlation between the changes of NH_4_^+^-N and its metabolites is relatively less. Since NH_4_^+^-N was directly related to NH_3_ emissions, we are more inclined to analyze the correlation between NH_3_ emissions and metabolites. We analyzed the data from the perspective of NH_3_ emissions. At the overall level, we found that the changes in the 4 metabolites were significantly correlated with NH_3_ emissions (*P* < 0.05). Among them, the metabolite with a positive correlation was metabolite No. 20, and the metabolites with a negative correlation were metabolites No. 31, 54, and 61. Among the above metabolites, only metabolite No. 31 was a consumable metabolite. This means that the synthesis of metabolites 54 and 61 increases, the synthesis of metabolite 20 decreases, and the consumption of metabolite 31 is less, which will realize the potential for NH_3_ emission reduction. As shown in Fig. 3c, we found that the dynamic changes of the above metabolites after the intervention of compound microalgal powder were closely related to NH_3_ emissions. We further explored the correlation between metabolites and NH_3_ emissions among each treatment group. We did not observe the significant correlation between metabolites No. 31, 54 and 61 and NH_3_ emissions in each treatment group, except that metabolite No. 20 maintained a significant and strong correlation (*r* = 0.9168, *P* = 0.0101) with NH_3_ emissions in group S. It is worth noting that although metabolite No. 35 was moderately correlated with emissions at the overall level (*r* = 0.3577, *P* = 0.0862), it remained significantly highly correlated (*r* = 0.9332, *P* = 0.0065), and the metabolite No. 61 was highly negatively correlated (*r* = −0.7924, *P* = 0.0602) with NH_3_ emissions in Group S. Combined with the analysis of Fig. 3c results, the above results provide data support for *S. platensis* compound microalgal powder (Group S) to reduce NH_3_ emissions by increasing the synthesis of metabolite 61 while reducing the synthesis of metabolites 20 and 35. We analyzed the key metabolites in the other two experimental groups. In group C, 3 metabolites were found to be closely related to NH_3_ emissions, namely metabolites No. 26 (*r* = −0.8895, *P* = 0.0176), No. 39 (*r* = 0.8602, *P* = 0.0279) and No. 48 (*r* = −0.8344, *P* = 0.0388). Among them, metabolite No. 39 was a consumable metabolite, while in group H, only metabolite No. 58 (*r* = 0.8301, *P* = 0.0409) was found to be the key consumable metabolite. Combined with the analysis of Fig. 3c results, the above results provide data support for the reduction of NH_3_ emissions by *C. vulgaris* compound microalgal powder (Group C) and *H. pluvialis* compound microalgal powder (Group H) respectively by increasing the synthesis of metabolite No. 26 and the consumption of metabolite No. 39, as well as the consumption of metabolite No. 58.

### Microalgal powder alters the changing trend of manure microbiota

**Fig. 5:**
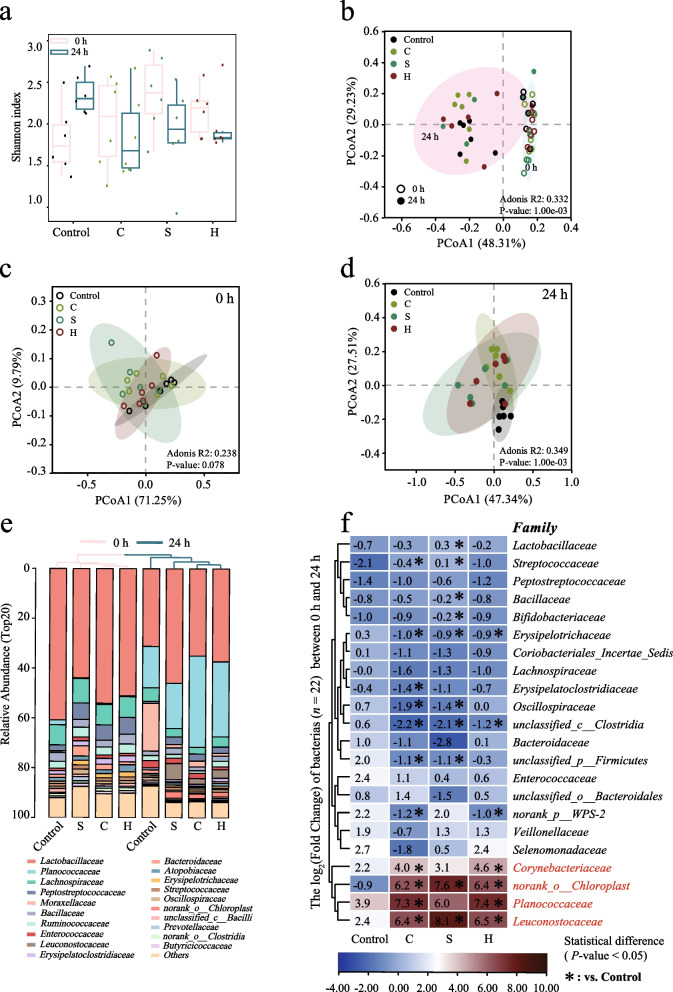
Microbial dynamic landscape. **a** Shannon index. **b**–**d** Principal component analysis (PCoA) plots based on the content of bacteria at the family level in manure with colors representing treatments. **e** Top 20 bacteria abundance at 0 h and 24 h. **f** Abundance change rates of 22 key bacteria between 0 and 24 h. An asterisk means *P* < 0.05

The changes in the manure microbiota were one of the main reasons affecting the transformation and flow of metabolites. Through amplicon sequencing, we found that the species diversity of fecal bacteria showed an increasing trend within 24 h, while the bacterial α diversity (Shannon index) decreased after the intervention of compound microalgal powder (Fig. 5a). We conducted cluster analysis on samples distributed at different times and found that the diversity of bacterial communities changed significantly over time (*P* < 0.05). We investigated the impact of natural changes during static storage on the manure microbiome (Fig. 5b). The two principal coordinates account for 29.23% and 48.31% of the total variation respectively. At the initial stage (0 h), there was no significant difference in the bacterial community diversity among the samples of each experimental group (Fig. 5c), but after 24 h, the samples of the microalgal powder intervention group gradually distinguished from those of the control group (Fig. 5d). The two principal coordinates account for 27.51% and 47.34% of the total variation respectively.

Therefore, we further explored the distribution of bacteria at the family level. Through cluster analysis, we reached a conclusion consistent with the previous one, that is, samples from different time periods were clustered separately (Fig. 5e). As shown in Fig. 5e, Lactobacillaceae, Erysipelotrichaceae, Peptostreptococcaceae, Bacillaceae, Ruminococcaceae and Lachnospiraceae dominated in the initial stage among the various treatment groups. The abundances all showed a downward trend after 24 h. On the contrary, the abundance of Planococcaceae shows an upward trend. Furthermore, after the intervention of compound microalgal powder, the abundances of Leuconostocaceae and norank_o_Chloroplast in manure were significantly higher than those in the control group after 24 h of static NH_3_ production, while the abundance of Moraxellaceae showed a downward trend compared with the control group (*P* < 0.05).

As shown in Fig. 4f, we present the abundance change ratio of the highly abundant family horizontal bacteria (*n* = 22) during the static NH_3_ production stage and conduct a significant difference analysis compared with the control group. Overall, we found that under the intervention of compound microalgal powder, the abundances of the 4 family-level bacteria showed a significant increase within 24 h. Among them, the change rates of norank_o_Chloroplast and Leuconostocaceae were significantly higher than those in the control group (*P* < 0.05). For Corynebacteriaceae and Planococcaceae, only the change rates of groups C and H were significantly higher than that of the control group (*P* < 0.05), while the change rate of group S was higher than that of the control group but not significant. On the other hand, after the intervention of compound microalgal powder, the rates of change of Erysipelotrichaceae and unclassified_c_Clostridia decreased significantly within 24 h (*P* < 0.05). In addition, the rates of change of Oscillospiraceae and unclassified_p_Firmicutes in groups C and S were significantly lower than those in the control group (*P* < 0.05), and similarly, the rate of change of norank_p_WPS-2 in groups C and H was significantly lower than that in the control group (*P* < 0.05). The above results indicate that the compound microalgal powder alters the bacterial community structure in manure, and the abundance of dominant bacteria (Corynebacteriaceae, Planococcaceae, Leuconostocaceae and norank_o_Chloroplast) significantly increases (*P* < 0.05).

### Key bacteria mediating changes in key metabolites related to NH_3_ emissions

**Fig. 6:**
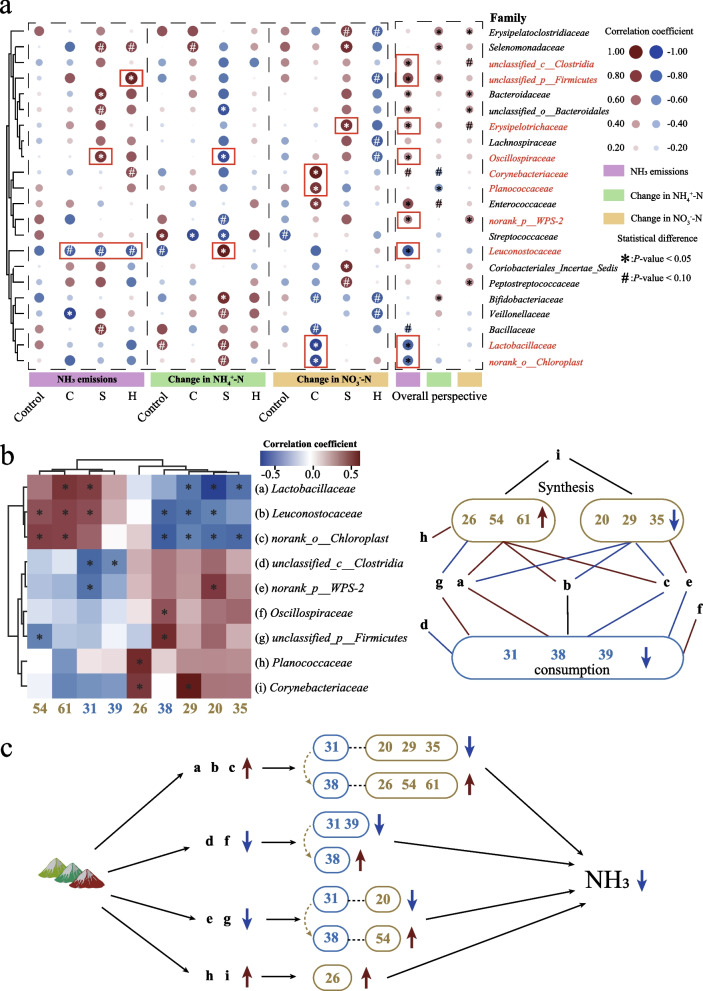
Microbiome-NH_3_ associations. **a** Correlation between the key bacteria, NH_3_ emissions, and inorganic nitrogen. An asterisk means *P* < 0.05 and the “#” indicates *P* < 0.10. **b** The association between bacteria and metabolites closely related to NH_3_ emissions. An asterisk means *P* < 0.05. **c** Proposed mechanism: Bacteria regulate nitrogen-containing metabolites to affect NH_3_ emissions. Green circles: synthetic metabolites; blue circles: consumable metabolites. Dark red arrows: increased synthesis/consumption; blue arrows: decreased synthesis/consumption

We also attempted to conduct a correlation analysis between the variations of these 22 family-level bacteria and the variations of NH_3_ emissions and inorganic nitrogen, and screened out the bacterial species closely related to the flow of nitrogen elements (Fig. 6a).

Similar to the results of GC/MS detection, most of the concerned bacterial species (13/22) were closely related to NH_3_ emissions. From the perspective of inorganic nitrogen, although it was found that the change in bacterial abundance was more closely related to NH_3_ emissions at the overall level, when compared among various treatment groups, we found that the change in NO_3_^−^-N content was more obvious. Among different types of compounds microalgal powder treatment groups, the bacterial species closely related to the changes in nitrate nitrogen content were not completely the same. In group C, the changes in the abundances of Corynebacteriaceae and Planococcaceae were significantly positively correlated with nitrate nitrogen (*P* < 0.05). Combined with the results of Fig. 5f, it means that the increase in the abundance of the above 2 bacteria mediated by the compound microalgal powder will limit the consumption of nitrate nitrogen. Combined with the overall level, the change in Corynebacteriaceae was positively correlated with NH_3_ emissions (*P* = 0.06542). It was speculated that the main pathways through which Corynebacteriaceae and Planococcaceae mediate the increase in NH_3_ emissions were to limit the consumption of NO_3_^−^-N and mitigate the accumulation of organic nitrogen. However, norank_o_Chloroplast and Lactobacillaceae were significantly negatively correlated with the change in NO_3_^−^-N and NH_3_ emissions (overall perspective) (*P* < 0.05). It is speculated that this bacterium has the function of mitigating NH_3_ production with the increase in the abundance of this bacterium, it promotes the conversion of NO_3_^−^-N to organic nitrogen and thereby reduces NH_3_ production. The Erysipelotrichaceae in group S was significantly positively correlated with the change in NO_3_^−^-N and NH_3_ emissions (overall perspective) (*P* < 0.05). It was speculated that this bacterium was an NH_3_-producing bacterium. With the intervention of compound microalgal powder, the abundance of this bacterium decreased significantly more in each experimental group than in the control group (Fig. 5f), thereby reducing NH_3_ emissions. Among the indicators of NH_4_^+^-N and NH_3_ emissions, we found that more significant correlations occurred in group S, which was related to the best NH_3_ emission reduction effect in Group S (*P* < 0.05). We found that Oscillospiraceae was significantly positively correlated with NH_3_ emissions, but significantly negatively correlated with changes in NH_4_^+^-N content, indicating that Oscillospiraceae were NH_3_-producing bacteria. Combined with the results of Fig. 5f, the abundance of this bacterium significantly decreased under the intervention of microalgal powder, promoting a reduction in NH_3_ emissions (*P* < 0.05). Similarly, a decrease in the abundance of unclassified_p_Firmicutes*, *unclassified_c_Clostridiaand*,* and Bacillaceae, and an increase in the abundance of Leuconostocaceae (Fig. 5f), the key bacteria that mitigate NH_3_ production, were also associated with reduced NH_3_ emissions. It mainly reduces NH_3_ emissions by promoting the deposition of NH_4_^+^-N into organic nitrogen.

We conducted a correlation analysis on the changes in the key family-level bacteria and metabolites discovered above, and the results were shown in Fig. 6b. Although metabolite No. 58 was previously defined as a key consumable metabolite in Fig. 4 (mainly found in group H), no significant correlation between it and key bacteria was found during the overall analysis (even when the correlation threshold was set at 0.3000). Similarly, we did not find any association between Erysipelotrichaceae and any key metabolites. We combined the heat map data with the previous definition of metabolites to draw a relationship graph between key microorganisms and the dynamic changes of metabolites (on the right side of the heat map). We found that Lactobacillaceae, Leuconostocaceae and norank_o_Chloroplast play important roles in regulating the dynamics of manure metabolism. As shown in Fig. 6c, after the intervention of the compound microalgal powder, the abundances of these 3 types of bacteria increased, and the associated consumption of metabolite 31 decreased while the synthesis of metabolites 20, 29, and 35 decreased. At this point, the consumption of metabolite 38 increased while the synthesis of metabolites 26, 54, and 61 increased, ultimately leading to a reduction in NH_3_ emissions. In addition, the reduced abundance of unclassified_c_Clostridia and Oscillospiraceae led to a decrease in the consumption of metabolites 31 and 39, while the consumption of metabolite 38 increased. The abundance of norank_p_WPS-2 and unclassified_p_Firmicutes decreased the consumption of metabolite 31 and the synthesis of metabolite 20, while increasing the consumption of metabolite 38 and the synthesis of metabolite 54. The functions of these 2 types of bacteria were similar to those of Leuconostocaceae*.* Although the abundance of Planococcaceae and Corynebacteriaceae increased significantly, it only promoted the synthesis of metabolites 26 and 29 (*P* < 0.05).

### Distribution characteristics of culturable NH_3_-producing bacteria

**Fig. 7:**
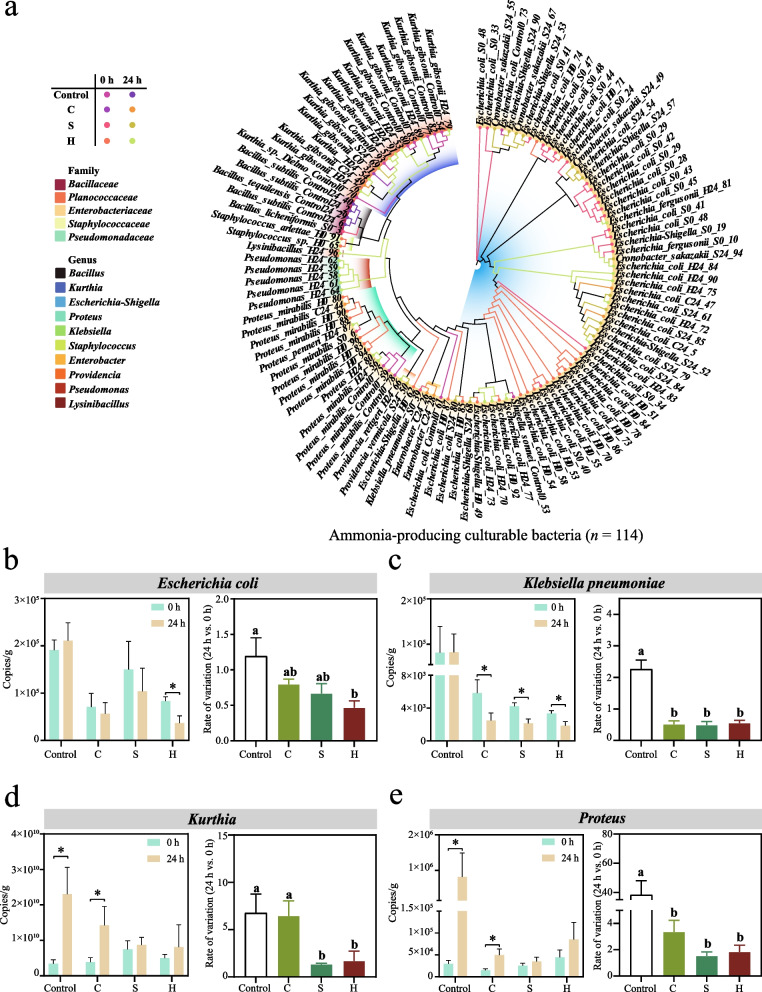
Identification and quantification of culturable bacteria. **a** Phylogenetic tree of isolates. **b**–**e** qPCR quantification of *Escherichia coli, Klebsiella pneumoniae, Kurthia*, and *Proteus. *Values are presented as mean ± SD. Different lowercase letters indicate significant differences among groups (*P* < 0.05, Tukey's test). *P < 0.05

We further isolated and identified the types of culturable NH_3_-producing bacteria in manure samples. A total of 114 culturable bacteria were obtained and a phylogenetic tree was constructed (Fig. 7a). These bacteria mainly belong to Enterobacteriaceae and Planococcaceae. In addition, we also obtained 3 other family-level bacteria, namely Bacillaceae, Staphylococcaceae and Pseudomonadaceae. The 114 culturable NH_3_-producing bacteria belong to 10 genera respectively, among these bacteria mainly were *Escherichia-Shigella* (*n* = 61), *Kurthia* (*n* = 17) and *Proteus* (*n* = 14). We found that among NH_3_-producing bacteria, *Escherichia coli* (*n* = 50) was the dominant strain, followed by *Kurthia gibsonii* (*n* = 15) and *Proteus mirabilis* (*n* = 12).

Next, we quantified the abundance of 4 culturable NH_3_-producing bacteria at the species level and genus level (Fig. 7b–e). The quantitative results indicated that after the intervention of the compound microalgal powder, the abundance of *Escherichia coli* and *Klebsiella pneumoniae* showed a downward trend after 24 h of static NH_3_ production stage. Among them, the abundance change rate (24 h vs. 0 h) of *Escherichia coli* in group H was significantly lower than that in the control group (*P* < 0.05; Fig. 7b). Meanwhile, the abundance of *Klebsiella pneumoniae* in all experimental groups at 24 h was significantly lower than that in the initial state, and the change rate was significantly lower than that in the control group (*P* < 0.05; Fig. 7c). It is also worth noting that after a 24 h static NH_3_ production stage, there was no significant difference in the abundance of *Escherichia coli* and *Klebsiella pneumoniae* in the control group. Although *Escherichia coli* is the main contributing bacteria among culturable NH_3_-producing bacteria, the differences among various treatment groups were not very common. Therefore, we supplemented the abundance change data of *Klebsiella pneumoniae*, which had the lowest proportion, and found very significant differences. The NH_3_ emission from manure may not be determined by the number of culturable NH_3_-producing bacteria. The subsequent quantitative results of the abundance of *Kurthia* and *Proteus* also confirmed this speculation (Fig. 7d and e). In the control group, the abundances of these NH_3_-producing bacteria significantly increased within 24 h, with the increase ratios reaching as high as 6.75 and 38.24 times respectively (*P* < 0.05). The increase in the abundances of these NH_3_-producing bacteria might be the main cause of NH_3_ emissions. After the intervention of compound microalgal powder, the abundances of these 2 bacteria in group C still increased significantly (*P* < 0.05), but there were no obvious changes in the other experimental groups. Meanwhile, the increase ratio of *Proteus* in each treatment group was significantly lower than that in the control group (*P* < 0.05; Fig. 7e). The above results were consistent with the previous finding that the addition of *Chlorella* compound microalgal powder had the poorest mitigatory effect on NH_3_ emissions (Fig. 1b and c). In conclusion, the compound microalgal powder achieves the effect of reducing NH_3_ emissions by mitigating the proliferation of NH_3_-producing bacteria or directly lowering their abundance.

## Discussion

### Microalgal powder reduces NH_3_ emissions by promoting the accumulation of organic nitrogen in manures

Studies have shown that the addition of microalgal powder to diets can effectively reduce the odor emissions from animal manure [37], but its mechanism of action is not yet clear. Whether the reduced NH_3_ is converted into NH_4_^+^-N, NO_3_^−^-N and N_2_O through the nitrogen cycle remains to be clarified. This study was the first to systematically explore the effects of the compound microalgal powder on NH_3_ emissions and nitrogen forms in laying hen manure. Although studies have shown that NH_3_ can be easily converted into NH_4_^+^-N and then into NO_3_^−^-N through nitrification to enter the nitrogen cycle [47], this study found that the inorganic nitrogen in the manure of the compound microalgal powder group was net consumed, which had no direct correlation with the reduction of NH_3_ emissions. It is worth noting that NO_3_^−^-N in the nitrogen cycle is transformed into greenhouse gas N_2_O under denitrification [48], but this study found that compound microalgal powder intervention significantly downregulated the expression of key genes for N_2_O generation, indicating that the traditional denitrification pathway is not the main cause of NH_3_ emission reduction (*P* < 0.05).

Nitrogen can not only be interconverted among inorganic nitrogen, but also be transformed into organic nitrogen through biological assimilation [49]. Through GC/MS analysis in this study, it was found that the intervention of compound microalgal powder promoted changes in the content of metabolites. After 24 h, nitrogen-containing organic matter in manure showed specific accumulation or consumption. The results of the correlation analysis show that compound microalgal powder achieves NH_3_ reduction by reshaping the nitrogen metabolism network and driving the dynamic transformation between inorganic nitrogen and organic nitrogen. The traditional NH_3_ emission reduction mechanisms mainly rely on chemical absorption and catalytic decomposition, such as NH_3_ decomposition through acidification absorption or photocatalysis [50, 51]. Feeding dietary supplements such as *Chlorella* and *Spirulina* can promote the chemical fixation of NH_3_ by reducing the pH value of manure [52]. This study found that feeding compound microalgal powder can regulate the nitrogen metabolism pathway in the manure of laying hens and achieve NH_3_ reduction by enhancing the bioconversion of inorganic nitrogen to organic nitrogen. This discovery provides a new mechanism analysis framework for microalgae emission reduction technology.

### Manure microbiota remodeling mediates the accumulation of organic nitrogen

Previous studies have shown that feeding microalgal powder can regulate the intestinal microecology of animals and affect the changes in the intestinal flora [53], which is related to the function of its own special functional substances that affect bacterial activity, such as the mitigation of *Escherichia coli* [54]. This study also reached a similar conclusion: under the intervention of compound microalgal powder (*H. pluvialis*), the abundance of *Escherichia coli* in manure decreased. The changes in manure microbiota are closely related to the intestinal microecology [55]. Feeding microalgal powder may regulate NH_3_ emissions by reshaping the manure microbiota.

This study discovered the pH regulation mediated by the proliferation of acid-producing bacterial communities. Amplification sequencing showed that compound microalgal powder intervention significantly increased the abundance of Corynebacteriaceae, Planococcaceae, Leuconostocaceae and norank_o_Chloroplast. These bacterial communities produce acidic substances such as glutamic acid, short-chain fatty acids (acetic acid and propionic acid), and lactic acid through metabolism [56–58], resulting in a decrease in manure pH and creating an acidic environment that is unfavorable for the emission of NH_3_ (*P* < 0.05). It is worth noting that Planococcaceae and Corynebacteriaceae have defects in NO_3_^−^-N metabolism capacity [59, 60], which may be the reason why they limit the consumption of NO_3_^−^-N and mitigate the accumulation of organic nitrogen. Their emission reduction effect is mainly attributed to the acid production mechanism rather than the transformation of nitrogen forms.

The Lactobacillaceae and Leuconostocaceae may be the main contributors mediating the remodeling of nitrogen metabolism. The increase in the abundances of these bacteria was significantly correlated with the dynamics of key metabolites in this study (*P* < 0.05). The Lactobacillaceae and Leuconostocaceae may promote the retention of organic nitrogen by altering the accumulation direction of organic nitrogen, such as reducing the synthesis of 3-Pentenenitrile, Quinoline, and 2H-1,2-Oxazine, then instead synthesizing Trideca-1,7,11-triene-1,1-dicarbonitrile, 2,3,5-Trimethyl-6-ethylpyrazine and 1,2,4-Triazolo[4,3-b]pyridazine. It may also influence the fate of NH_3_ emissions by changing the consumption pattern of nitrogen-containing substrates, such as reducing the consumption of 1H-Pyrrole-2,5-dione and instead utilizing hexanenitrile. Metabolite 54 is commonly found in fermentation products and microbial fermentation products. It is a compound with a special aroma produced by microorganisms during nitrogen metabolism [61]. The above process is related to the synergistic effect of characteristics such as nitrite reductase and lactein [62], such as creating an acidic environment to mitigate the activity of NH_3_-producing bacteria, enhancing the accumulation of organic nitrogen and promoting the interaction of bacterial communities to accelerate the transformation and accumulation of organic nitrogen [63].

Fed compound microalgal powder may have an mitigatory effect on NH_3_-producing bacterial communities. This study found that the intervention of compound microalgal powder significantly reduced the abundance of Erysipelotrichaceae and Oscillospiraceae (*P* < 0.05). Previous studies have shown that an increase in ammonia emissions is often accompanied by an up-regulation of the abundances of Erysipelotrichaceae and Oscillospiraceae [64, 65]. This study also found that these bacteria have a significant positive correlation with NH_3_ emissions. At the same time, with a decrease in their abundances, the consumption of 1H-Pyrrole-2,5-dione and 1H-Pyrazole decreases. The decrease in its abundance directly weakens the ammonification process and forms a dual mitigatory network with the aforementioned acid-producing bacterial community.

### The NH_3_ emission reduction mechanism mediated by microalgal

Although this study found that all the compound algal powder treatment groups had the potential for NH_3_ emission reduction, there were differences among all the treatment groups. The complex algal powder mainly composed of *S. platensis* showed a stronger potential for NH_3_ emission reduction. Through amplicon sequencing analysis in this study, it was found that *S. platensis* intervention specifically increased the abundance of Leuconostocaceae and simultaneously mitigated the proliferation of Oscillospiraceae in laying hen manure. This result forms a cross-species verification with the related studies on broilers and Holstein–Friesian dairy cattle [37, 66], and the phenomenon of NH_3_ emission reduction caused by the addition of *S. platensis* is universal. The increase in the synthesis of nitrogen-containing aromatic compounds was significantly positively correlated with the abundance of Leuconostocaceae (*r* = 0.4884, *P* < 0.001), and this metabolite directly reduced the NH_3_ emission by enhancing the biological retention efficiency of organic nitrogen. It has been reported in studies that phycocyanin, a characteristic component of *S. platensis*, has antibacterial effects and shows dose-dependent mitigation on Gram-negative bacteria, such as *Proteus*, *Escherichia coli*, and *Klebsiella pneumoniae* [67, 68]. Similar results were obtained in this study: after feeding *S. platensis* compound microalgal powder, the proliferation of *Escherichia coli* and *Klebsiella pneumoniae* was mitigated. Its mechanism of action may be related to the activity of synthase in the cell wall [69, 70].

The *C. vulgaris* lacks the unique antibacterial components such as phycocyanin in *S. platensis* and has a weak mitigatory ability against NH_3_-producing bacteria (such as Oscillospiraceae). Currently, the functional research on *C. vulgaris* mainly focuses on wastewater purification [71]. Although astaxanthin specifically synthesized by *H. pluvialis* has antioxidant effects, which can reduce oxidative stress in animals and thereby improve intestinal health and shape a stable microecological environment [38], there are currently not many studies indicating that the improvement of antioxidant capacity is directly related to NH_3_ emissions. While *C. vulgaris* and *H. pluvialis* exhibit NH_3_ emission reduction potential similar to *S. platensis*, current evidence suggests their mechanisms remain comparatively limited, primarily through nitrogen assimilation and antioxidant activity. This study demonstrates that combinatorial supplementation of these functional microalgae species effectively reduces NH_3_ emissions in laying hen manure (*P* < 0.05), with all tested microalgal formulations showing significant mitigation effects. These findings highlight the necessity for deeper mechanistic exploration of interspecific microalgal synergies while providing strategic insights for developing multifunctional microalgae-based feed formulations.

### Limitation and future scope

This study utilized technologies such as GC/MS and amplicon sequencing to reveal the potential mechanism of compound microalgal powder regulating NH_3_ emissions from laying hen manure. However, its practical application still faces the following challenges: Firstly, although the short-term (24 h) assessment based on the static NH_3_ production system shows a significant emission reduction effect, it does not simulate the influence of dynamic ventilation and seasonal fluctuations in industrial chicken houses on NH_3_ volatilization, which may overestimate the long-term efficacy of microalgal powder. Secondly, the screening of key functional microbiota relies on statistical inference. While culturable bacterial data provide preliminary support, the absence of direct validation via microbial colonization assays may introduce biases in the screening of certain key microbial taxa. Furthermore, the biosafety and economic viability of microalgal powder additives remain ill-defined. They may also precipitate management challenges in downstream processes, such as the potential risks of antibiotic resistance gene (ARG) transmission, or the presence of fertilizer efficiency advantages in the manure derived from laying hens fed with microalgal powder.

Based on the above limitations, future research should focus on the following directions: Firstly, focus on the in-depth analysis of the mechanism: integrate multiple omics (metagenomics, transcriptomics, targeted metabolomics, etc.), construct a complete regulatory network for the compound microalgal powder to regulate the bacterial flora and affect the flow of metabolites, thereby achieving NH_3_ emission reduction, and use machine learning to establish a prediction model between the content of metabolites and the emission reduction efficiency to optimize the dosage of microalgal powder and clarify the critical threshold of the emission reduction efficiency. Then, focus on the assessment of biosafety: by monitoring the microbial adaptability (nitrogen metabolism adaptability) and biosafety risks (transmission of ARG) under the long-term intervention of microalgal powder, evaluate the ecological domestication effect. Next, considering the circular economy and cross-species universality: further explore the utilization of aquaculture wastewater for microalgae breeding to achieve feed utilization, promote the recycling of nitrogen resources, and verify the universality of microalgal powder in cross-species such as ruminants. Finally, quantify the comprehensive benefits: carry out the full life cycle analysis to quantify the reduction of nitrogen footprint and environmental economic benefits throughout the entire chain from aquaculture wastewater to microalgae breeding, algae powder feeding to laying hens, and laying hen manure returning to the field for utilization, and build a sustainable agricultural framework for nitrogen reduction and resource recycling.

## Conclusion

This study found that the compound algal powder composed of *Chlorella vulgaris*, *Spirulina platensis* and *Haematococcus pluvialis*, when fed to laying hens, all had the potential to reduce ammonia emissions in their manure, with a reduction of 40.47%–77.84%. Compound microalgal powder alters the evolution pattern of manure bacterial communities, proliferates acid-producing bacteria, reduces the abundance of NH_3_-producing bacteria, promotes the conversion of inorganic nitrogen to organic nitrogen and regulates the manure nitrogen metabolism network, thereby increasing the synthesis of non-ammonia volatile substances (such as 2,3,5-trimethyl-6-ethylpyrazine). This study provides a new analytical framework for the NH_3_ emission reduction mechanism of microalgae feed and offers a theoretical basis for the development of environmentally friendly feed.

## Supplementary Information

Additional file 1: Table S1. The nutritional composition of microalgae powder. Table S2. Composition (ingredients, nutrients) of the experimental basal feeds. Table S3. The primer sequences of target bacteria in this paper. Table S4. The primer sequences of nitrogen cycling-related genes in this paper. Fig. S1. Change rate of expression levels of nitrogen cycling gene (24 h vs. 0 h).

## Data Availability

Data will be made available on request.

## Abbreviations

ARG: Antibiotic resistance gene
*C. vulgaris*: *Chlorella vulgaris*
GC-MS: Gas chromatography-mass spectrometry
*H. pluvialis*: *Haematococcus pluvialis*
NH_3_: Ammonia
NH_4_^+^-N: Ammonium nitrogen
NO_3_^–^-N: Nitrate nitrogen
N_2_O: Nitrous oxide
qPCR: Quantitative polymerase chain reaction
qRT-PCR: Quantitative reverse transcription polymerase chain reaction
*S. platensis*: *Spirulina platensis*

## References

[1] Yan X, Ying Y, Li K, Zhang Q, Wang K. A review of mitigation technologies and management strategies for greenhouse gas and air pollutant emissions in livestock production. J Environ Manage. 2024;352:120028. 10.1016/j.jenvman.2024.120028. 38219668 10.1016/j.jenvman.2024.120028

[2] Heederik D, Sigsgaard T, Thorne PS, Kline JN, Avery R, Bonlokke JH, et al. Health effects of airborne exposures from concentrated animal feeding operations. Environ Health Perspect. 2007;115(2):298–302. 10.1289/ehp.8835. 17384782 10.1289/ehp.8835 PMC1817709

[3] Alfonso-Avila AR, Cirot O, Lambert W, Letourneau-Montminy MP. Effect of low-protein corn and soybean meal-based diets on nitrogen utilization, litter quality, and water consumption in broiler chicken production: insight from meta-analysis. Animal. 2022;16(3):10. 10.1016/j.animal.2022.100458. 10.1016/j.animal.2022.100458 35183011

[4] Deng Z, Geng X, Shi M, Chen X, Wei Z. Effect of different moisture contents on hydrogen sulfide malodorous gas emission during composting. Bioresour Technol. 2023;380:129093. 10.1016/j.biortech.2023.129093. 37100296 10.1016/j.biortech.2023.129093

[5] Ritz CW, Fairchild BD, Lacy MP. Implications of ammonia production and emissions from commercial poultry facilities: a review. J Appl Poult Res. 2004;13(4):684–92. 10.1093/japr/13.4.684.

[6] Bailey MA, Hess JB, Krehling JT, Macklin KS. Effects of feed-through sulfur on growth performance, atmospheric ammonia levels, and footpad lesions in broilers raised beginning with built-up litter. Animals. 2022;12(17):2206. 10.3390/ani12172206. 10.3390/ani12172206 PMC9454838 36077926

[7] Naseem S, King AJ. Ammonia production in poultry houses can affect health of humans, birds, and the environment-techniques for its reduction during poultry production. Environ Sci Pollut Res. 2018;25(16):15269–93. 10.1007/s11356-018-2018-y. 10.1007/s11356-018-2018-y 29705898

[8] Liao W, Liu M, Huang X, Wang T, Xu Z, Shang F, et al. Estimation for ammonia emissions at county level in China from 2013 to 2018. Sci China Earth Sci. 2022;65(6):1116–27. 10.1007/s11430-021-9897-3.

[9] Wang H, Zhao Z, Winiwarter W, Bai Z, Wang X, Fan X, et al. Strategies to reduce ammonia emissions from livestock and their cost-benefit analysis: a case study of sheyang county. Environ Pollut. 2021;290:118045. 10.1016/j.envpol.2021.118045. 34488163 10.1016/j.envpol.2021.118045

[10] Yu Q, Ge X, Zheng H, Xing J, Duan L, Lv D, et al. A probe into the acid deposition mitigation path in China over the last four decades and beyond. Natl Sci Rev. 2024;11(4):nwae7. 10.1093/nsr/nwae007. 10.1093/nsr/nwae007 PMC10941815 38495813

[11] Liu L, Zhang X, Xu W, Liu X, Zhang Y, Li Y, et al. Fall of oxidized while rise of reduced reactive nitrogen deposition in China. J Clean Prod. 2020;272:10. 10.1016/j.jclepro.2020.122875.

[12] Rosa E, Arriaga H, Merino P. Strategies to mitigate ammonia and nitrous oxide losses across the manure management chain for intensive laying hen farms. Sci Total Environ. 2022;803:150017. 10.1016/j.scitotenv.2021.150017. 34500278 10.1016/j.scitotenv.2021.150017

[13] Namroud NF, Shivazad M, Zaghari M. Effects of fortifying low crude protein diet with crystalline amino acids on performance, blood ammonia level, and excreta characteristics of broiler chicks. Poult Sci. 2008;87(11):2250–8. 10.3382/ps.2007-00499. 18931175 10.3382/ps.2007-00499

[14] Rehman HU, Vahjen W, Awad WA, Zentek J. Indigenous bacteria and bacterial metabolic products in the gastrointestinal tract of broiler chickens. Arch Anim Nutr. 2007;61(5):319–35. 10.1080/17450390701556817. 18030916 10.1080/17450390701556817

[15] Steenfeldt S, Nielsen BL. Welfare of organic laying hens kept at different indoor stocking densities in a multi-tier aviary system. II: live weight, health measures and perching. Animal. 2015;9(9):1518–28. 10.1017/S1751731115000725. 10.1017/S1751731115000725 25990629

[16] Kim WK, Patterson PH. Production of an egg yolk antibody specific to microbial uricase and its inhibitory effects on uricase activity. Poult Sci. 2003;82(10):1554–8. 10.1093/ps/82.10.1554. 14601732 10.1093/ps/82.10.1554

[17] Zhang C, Geng X, Wang H, Zhou L, Wang B. Emission factor for atmospheric ammonia from a typical municipal wastewater treatment plant in south China. Environ Pollut. 2017;220:963–70. 10.1016/j.envpol.2016.10.082. 27823866 10.1016/j.envpol.2016.10.082

[18] Zhao H, Li S, Pu J, Wang H, Dou X. Effects of *Bacillus*-based inoculum on odor emissions co-regulation, nutrient element transformations and microbial community tropological structures during chicken manure and sawdust composting. J Environ Manage. 2024;354:120328. 10.1016/j.jenvman.2024.120328. 10.1016/j.jenvman.2024.120328 38354615

[19] Huang J, Yu Z, Gao H, Yan X, Chang J, Wang C, et al. Chemical structures and characteristics of animal manures and composts during composting and assessment of maturity indices. PLoS One. 2017;12(6):e178110. 10.1371/journal.pone.0178110. 10.1371/journal.pone.0178110 PMC5467826 28604783

[20] Huang Y, Chen Y, Huang H, Shah GM, Lin J, Yan M, et al. Hyperthermophilic pretreatment composting can reduce ammonia emissions by controlling proteolytic bacterial community and the physicochemical properties. Bioresour Bioprocess. 2023. 10.1186/s40643-023-00659-y. 38647615 10.1186/s40643-023-00659-y PMC10992325

[21] Zeng Y, De Guardia A, Ziebal C, De Macedo FJ, Dabert P. Nitrification and microbiological evolution during aerobic treatment of municipal solid wastes. Bioresour Technol. 2012;110:144–52. 10.1016/j.biortech.2012.01.135. 22342082 10.1016/j.biortech.2012.01.135

[22] Whitehead TR, Cotta MA. Isolation and identification of hyper-ammonia producing bacteria from swine manure storage pits. Curr Microbiol. 2004;48(1):20–6. 10.1007/s00284-003-4084-7. 15018098 10.1007/s00284-003-4084-7

[23] Doolittle M, Raina A, Lax A, Boopathy R. Presence of nitrogen fixing *Klebsiella pneumoniae* in the gut of the formosan subterranean termite (*Coptotermes formosanus*). Bioresour Technol. 2008;99(8):3297–300. 10.1016/j.biortech.2007.07.013. 10.1016/j.biortech.2007.07.013 17709245

[24] Krober TF, Kulling DR, Menzi H, Sutter F, Kreuzer M. Quantitative effects of feed protein reduction and methionine on nitrogen use by cows and nitrogen emission from slurry. J Dairy Sci. 2000;83(12):2941–51. 10.3168/jds.S0022-0302(00)75194-0. 11132866 10.3168/jds.S0022-0302(00)75194-0

[25] Ma Y, Hou Y, Zhang T, Zhu X, Fang Q, Oenema O. Decreasing environmental footprints of dairy production systems through optimization of feed rations and origins. J Clean Prod. 2024;461:142637. 10.1016/j.jclepro.2024.142637.

[26] Greenhalgh S, Chrystal PV, Selle PH, Liu SY. Reduced-crude protein diets in chicken-meat production: justification for an imperative. Worlds Poult Sci J. 2020;76(3):537–48. 10.1080/00439339.2020.1789024.

[27] Tang JW, Sun H, Yao XH, Wu YF, Wang X, Feng J. Effects of replacement of soybean meal by fermented cottonseed meal on growth performance, serum biochemical parameters and immune function of yellow-feathered broilers. Asian-Australas J Anim Sci. 2012;25(3):393–400. 10.5713/ajas.2011.11381. 25049578 10.5713/ajas.2011.11381 PMC4092957

[28] Tang Y, Yang F, Pang H, Zhao S, Ma H, Li H, et al. Assessing the feasibility of adjusting the dry matter content utilizing corn grits and cottonseed meal and inoculating with *Lactiplantibacillus plantarum* in the production of fermented feed from kitchen waste. Process Saf Environ Prot. 2024;192:129–38. 10.1016/j.psep.2024.10.032.

[29] Ahirwar A, Kesharwani K, Deka R, Muthukumar S, Khan MJ, Rai A, et al. Microalgal drugs: a promising therapeutic reserve for the future. J Biotechnol. 2022;349:32–46. 10.1016/j.jbiotec.2022.03.012. 35339574 10.1016/j.jbiotec.2022.03.012

[30] de Souza MP, Hoeltz M, Gressler PD, Benitez LB, Schneider RCS. Potential of microalgal bioproducts: general perspectives and main challenges. Waste Biomass Valorization. 2019;10(8):2139–56. 10.1007/s12649-018-0253-6.

[31] Van Nerom S, Coleman B, De Baets R, Van Immerseel F, Robbens J, Delezie E. Microalgae as feed additives in poultry: a review on the health-promoting effects. Algal Res. 2024;83:103733. 10.1016/j.algal.2024.103733.

[32] Hao T, Balamurugan S, Zhang Z, Liu S, Wang X, Li D, et al. Effective bioremediation of tobacco wastewater by microalgae at acidic pH for synergistic biomass and lipid accumulation. J Hazard Mater. 2022;426:127820. 10.1016/j.jhazmat.2021.127820. 34865896 10.1016/j.jhazmat.2021.127820

[33] Colusse GA, Carneiro J, Duarte MER, Carvalho JCD, Noseda MD. Advances in microalgal cell wall polysaccharides: a review focused on structure, production, and biological application. Crit Rev Biotechnol. 2022;42(4):562–77. 10.1080/07388551.2021.1941750. 34320897 10.1080/07388551.2021.1941750

[34] Andriopoulos V, Kornaros M. Microalgal phenolics: systematic review with a focus on methodological assessment and meta-analysis. Mar Drugs. 2024;22(10):460. 10.3390/md22100460. 39452869 10.3390/md22100460 PMC11509163

[35] Wang X, Li S, Chen Y, Chen Y, Guo J, Liu S, et al. New insights into microalgal photobiological hydrogen production: potential role of aggregation forms in modulating the hydrogenase activity and metabolic properties of microalgae during hydrogen production. Chem Eng J. 2024;500:156802. 10.1016/j.cej.2024.156802.

[36] Yang M, Liu Z, Wang A, Nopens I, Hu H, Chen H. High biomass yields of *chlorella protinosa* with efficient nitrogen removal from secondary effluent in a membrane photobioreactor. J Environ Sci. 2024;146:272–82. 10.1016/j.jes.2023.10.036. 10.1016/j.jes.2023.10.036 38969455

[37] Park JH, Lee S, Kim IH. Effect of dietary Spirulina (*Arthrospira*) *platensis* on the growth performance, antioxidant enzyme activity, nutrient digestibility, cecal microflora, excreta noxious gas emission, and breast meat quality of broiler chickens. Poult Sci. 2018;97(7):2451–9. 10.3382/ps/pey093. 10.3382/ps/pey093 29672750

[38] Jeong JS, Kim IH. Effect of astaxanthin produced by *Phaffia rhodozyma* on growth performance, meat quality, and fecal noxious gas emission in broilers. Poult Sci. 2014;93(12):3138–44. 10.3382/ps.2013-03847. 10.3382/ps.2013-03847 25260529

[39] Saeed M, Babazadeh D, Naveed M, Alagawany M, El-Hack MEA, Arain MA, et al. In ovo delivery of various biological supplements, vaccines and drugs in poultry: current knowledge. J Sci Food Agric. 2019;99(8):3727–39. 10.1002/jsfa.9593. 30637739 10.1002/jsfa.9593

[40] Dinika I, Verma DK, Balia R, Utama GL, Patel AR. Potential of cheese whey bioactive proteins and peptides in the development of antimicrobial edible film composite: a review of recent trends. Trends Food Sci Technol. 2020;103:57–67. 10.1016/j.tifs.2020.06.017.

[41] Alagawany M, Elnesr SS, Farag MR, Abd El-Hack ME, Barkat RA, Gabr AA, et al. Potential role of important nutraceuticals in poultry performance and health - a comprehensive review. Res Vet Sci. 2021;137:9–29. 10.1016/j.rvsc.2021.04.009. 33915364 10.1016/j.rvsc.2021.04.009

[42] Liu S, Shi L, Luo H, Chen K, Song M, Wu Y, et al. Processed microalgae: green gold for tissue regeneration and repair. Theranostics. 2024;14(13):5235–61. 10.7150/thno.99181. 39267781 10.7150/thno.99181 PMC11388063

[43] Silva-Neto JF, Nunes AJP, Sabry-Neto H, Sá MVC. Spirulina meal has acted as a strong feeding attractant for litopenaeus vannamei at a very low dietary inclusion level. Aquac Res. 2012;43(3):430–7. 10.1111/j.1365-2109.2011.02846.x.

[44] Liu B, Jiang J, Yu D, Lin G, Xiong YL. Effects of supplementation of microalgae (*Aurantiochytrium* sp.) to laying hen diets on fatty acid content, health lipid indices, oxidative stability, and quality attributes of meat. Foods. 2020;9(9):1271. 10.3390/foods9091271. 10.3390/foods9091271 PMC7555786 32927865

[45] Costa MM, Spínola MP, Prates JAM. Microalgae as an alternative mineral source in poultry nutrition. Vet Sci. 2024;11(1):44. 10.3390/vetsci11010044. 38275926 10.3390/vetsci11010044 PMC10819150

[46] Zhao D, Gao L, Gong F, Feng J, Zhang H, Wu S, et al. Tmt-based quantitative proteomic analysis reveals eggshell matrix protein changes correlated with eggshell quality in jing tint 6 laying hens of different ages. Poult Sci. 2024;103(3):103463. 10.1016/j.psj.2024.103463. 38281332 10.1016/j.psj.2024.103463 PMC10840124

[47] Lindeboom REF, Ilgrande C, Carvajal-Arroyo JM, Coninx I, Van Hoey O, Roume H, et al. Nitrogen cycle microorganisms can be reactivated after space exposure. Sci Rep. 2018;8:13783. 10.1038/s41598-018-32055-4. 10.1038/s41598-018-32055-4 PMC6137101 30214003

[48] Bonin P, Tamburini C, Michotey V. Determination of the bacterial processes which are sources of nitrous oxide production in marine samples. Water Res. 2002;36(3):722–32. 10.1016/s0043-1354(01)00269-x. 11827333 10.1016/s0043-1354(01)00269-x

[49] Zhao M, Liu D, Zhou J, Wei Z, Wang Y, Zhang X. Ammonium stress promotes the conversion to organic nitrogen and reduces nitrogen loss based on restructuring of bacterial communities during sludge composting. Bioresour Technol. 2022;360:127547. 10.1016/j.biortech.2022.127547. 35777648 10.1016/j.biortech.2022.127547

[50] Liu J, Li X, Xu Y, Wu Y, Wang R, Zhang X, et al. Highly efficient reduction of ammonia emissions from livestock waste by the synergy of novel manure acidification and inhibition of ureolytic bacteria. Environ Int. 2023;172:107768. 10.1016/j.envint.2023.107768. 36709675 10.1016/j.envint.2023.107768

[51] Zhao Z, Zhang M, Wu Y, Song W, Yan J, Qi X, et al. Ammonia energy: synthesis and utilization. Ind Eng Chem Res. 2024;63(18):8003–24. 10.1021/acs.iecr.4c00384.

[52] Martínez Y, Ayala L, Hurtado C, Más D, Rodríguez R. Effects of dietary supplementation with red algae powder (*Chondrus crispus*) on growth performance, carcass traits, lymphoid organ weights and intestinal pH in broilers. Braz J Poult Sci. 2019. 10.1590/1806-9061-2019-1015.

[53] Al-Khalaifah HS, Al-Nasser A, Surrayai T. Effects from dietary addition of *Sargassum* sp., *Spirulina* sp., or *Gracilaria* sp. Powder on immune status in broiler chickens. Front. Vet. Sci. 2022;9:928235. 10.3389/fvets.2022.928235. 10.3389/fvets.2022.928235 PMC9234524 35769316

[54] Occhipinti PS, Russo N, Foti P, Pino A, Randazzo CL, Pollio A, et al. An indigenous microalgal pool containing *Klebsormidium* sp. K39 as a stable and efficacious biotechnological strategy forescherichia coli removal in urban wastewater treatment. J Sci Food Agric. 2025;105(2):1288–97. 10.1002/jsfa.13918. 10.1002/jsfa.13918 PMC11632170 39310998

[55] Hou S, Yu J, Li Y, Zhao D, Zhang Z. Advances in fecal microbiota transplantation for gut dysbiosis-related diseases. Adv Sci. 2025;12(13):2413197. 10.1002/advs.202413197. 10.1002/advs.202413197 PMC11967859 40013938

[56] Fusco W, Lorenzo MB, Cintoni M, Porcari S, Rinninella E, Kaitsas F, et al. Short-chain fatty-acid-producing bacteria: key components of the human gut microbiota. Nutrients. 2023;15(9):2211. 10.3390/nu15092211. 37432351 10.3390/nu15092211 PMC10180739

[57] Zuchowski R, Schito S, Mack C, Wirtz A, Bott M, Polen T, et al. Ale reveals a surprising link between [Fe-S] cluster formation, tryptophan biosynthesis and the potential regulatory protein TrpP in *Corynebacterium glutamicum*. BMC Microbiol. 2025;25:214. 10.1186/s12866-025-03939-z. 10.1186/s12866-025-03939-z PMC11995493 40229682

[58] Kim MJ, Jeong JY, Hwang IM, Lee J. Modulation of fermentation dynamics in kimchi using *Leuconostoc mesenteroides* starter. Food Biosci. 2025;66:106317. 10.1016/j.fbio.2025.106317.

[59] Gupta RS, Patel S. Robust demarcation of the family Caryophanaceae (Planococcaceae) and its different genera including three novel genera based on phylogenomics and highly specific molecular signatures. Front Microbiol. 2020;10:2821. 10.3389/fmicb.2019.02821. 10.3389/fmicb.2019.02821 PMC6971209 32010063

[60] Platzen L, Koch-Koerfges A, Weil B, Brocker M, Bott M. Role of flavohaemoprotein Hmp and NarGHJI reductase narghji of *Corynebacterium**glutamicum* for coping with nitrite and nitrosative stress. FEMS Microbiol Lett. 2014;350(2):239–48. 10.1111/1574-6968.12318. 10.1111/1574-6968.12318 24237595

[61] Hao Y, Wang Z, Zou Y, He R, Ju X, Yuan J. Effect of static-state fermentation on volatile composition in rapeseed meal. J Sci Food Agric. 2020;100(5):2145–52. 10.1002/jsfa.10238. 31903609 10.1002/jsfa.10238

[62] Wang L, Huang J, Hu S, Li X, Zhang Y, Cheng W, et al. The dynamic changes and correlations between biochemical properties, flavor and microbial community during fermentation of asparagus by-products. Food Chem. 2025;463:141173. 10.1016/j.foodchem.2024.141173. 39276550 10.1016/j.foodchem.2024.141173

[63] Boasiako TA, Ekumah J, Yaqoob S, Aregbe AY, Li Y, Ashiagbor K, et al. Synergistic effects of *Lactobacillus* strains and *Acetobacter pasteurianus* on jujube puree’s product functionality and quality. Heliyon. 2024;10(2):e24447. 10.1016/j.heliyon.2024.e24447. 10.1016/j.heliyon.2024.e24447 PMC10826817 38293436

[64] Ortiz-Chura A, Gere J, Marcoppido G, Depetris G, Cravero S, Faverín C, et al. Dynamics of the ruminal microbial ecosystem, and inhibition of methanogenesis and propiogenesis in response to nitrate feeding to holstein calves. Anim Nutr. 2021;7(4):1205–18. 10.1016/j.aninu.2021.07.005. 34754962 10.1016/j.aninu.2021.07.005 PMC8556761

[65] Dong J, Zhao Z, Wang Z, Li S, Zhang Y, Sun Z, et al. Impact of deoxynivalenol on rumen function, production, and health of dairy cows: insights from metabolomics and microbiota analysis. J Hazard Mater. 2024;465:133376. 10.1016/j.jhazmat.2023.133376. 38159518 10.1016/j.jhazmat.2023.133376

[66] Lobo RR, Siregar MU, Da Silva SS, Monteiro AR, Salas-Solis G, Vicente ACS, et al. Partial replacement of soybean meal with microalgae biomass on in vitro ruminal fermentation may reduce ruminal protein degradation. J Dairy Sci. 2024;107(3):1460–71. 10.3168/jds.2023-24016. 37944802 10.3168/jds.2023-24016

[67] Yu Z, Zhao W, Sun H, Mou H, Liu J, Yu H, et al. Phycocyanin from microalgae: a comprehensive review covering microalgal culture, phycocyanin sources and stability. Food Res Int. 2024;186:114362. 10.1016/j.foodres.2024.114362. 38729724 10.1016/j.foodres.2024.114362

[68] Furmaniak MA, Misztak AE, Franczuk MD, Wilmotte A, Waleron M, Waleron KF. Edible cyanobacterial genus *Arthrospira*: actual state of the art in cultivation methods, genetics, and application in medicine. Front Microbiol. 2017. 10.3389/fmicb.2017.02541. 10.3389/fmicb.2017.02541 PMC5741684 29326676

[69] El-Mohsnawy E, Abu-Khudir R. A highly purified c-phycocyanin from thermophilic cyanobacterium *Thermosynechococcus elongatus* and its cytotoxic activity assessment using an in vitro cell-based approach. J Taibah Univ Sci. 2020;14(1):1218–25. 10.1080/16583655.2020.1812287.

[70] Arindita NPY, Munawaroh HSH, Aisyah S, Khoiriah SF. Antibacterial potential of *Spirulina**platensis* phycocyanin peptides: a multiple molecular docking analysis. J Bioteknol Biosains Indones. 2023;10(2):312–30.

[71] Rajamanickam R, Selvasembian R. Insights into the potential of chlorella species in the treatment of hazardous pollutants from industrial effluent. World J Microbiol Biotechnol. 2025. 10.1007/s11274-025-04351-5. 40232538 10.1007/s11274-025-04351-5

