# Supplementary material for: Practical microalgal supplementation: reducing ammonia emission from manure in commercial layer production
Source: J Anim Sci Biotechnol. 2025 Oct 27;16:140. doi: 10.1186/s40104-025-01264-z (PMC12557985; doi:10.1186/s40104-025-01264-z)
Supplement: Supplementary file 1 — Additional file 1: Table S1. The nutritional composition of microalgae powder. Table S2. Composition (ingredients, nutrients) of the experimental basal feeds. Table S3. The primer sequences of target bacteria in this paper. Table S4. The primer sequences of nitrogen cycling-related genes in this paper. Fig. S1. Change rate of expression levels of nitrogen cycling gene (24 h vs. 0 h). [file 40104_2025_1264_MOESM1_ESM.docx]

**Table S1** The nutritional composition of microalgae powder

| **Nutrient composition, g/100g microalgae** | ***Chlorella vulgaris*** | ***Spirulina platensis*** | ***Haematococcus pluvialis*** |
| --- | --- | --- | --- |
| Water content | 5.74 | 3.89 | 3.56 |
| Crude protein | 52.87 | 70.99 | 30.16 |
| Crude fat | 0.90 | 1.29 | 18.83 |
| Crude ash | 4.08 | 6.23 | 10.11 |
| Crude fiber | 3.02 | < 1 | < 1 |
| NDF^1^ | 0.20 | 30.23 | 16.57 |
| ADF^2^ | 1.50 | 1.39 | 1.60 |
| Total carotenoids | / | / | 1.45 |
| Red pigment | / | / | 2.05 |
| GE^3^，kcal/100g DM^4^ | 441.3 | 479.5 | 494.4 |

^1^*NDF* Neutral detergent fibre

^2^*ADF* Acid detergent fibre

^3^*GE* Gross energy

^4^*DM* Dry matter

**Table S2** Composition (ingredients, nutrients) of the experimental basal feeds

| **Items** | **Composition, %** |
| --- | --- |
| Ingrediet composition, % |  |
| Corn | 62.00 |
| Soyabean | 22.00 |
| Limestone | 9.00 |
| Soybean oil | 1.20 |
| Wheat bran | 2.50 |
| Premix^1^ | 3.30 |
| Total | 100 |
| Nutrient composition, % |  |
| Crude protein | 15.48 |
| Crude fat | 4.18 |
| Crude fiber | 4.00 |
| Lysine | 0.60 |
| Methionine | 0.36 |
| Calcium | 3.90 |
| Total phosphorus | 0.50 |

^1^Premix: The vitamin-mineral premix supplied the following per kilogram of complete feed: vitamin A, 8,000 IU; vitamin D₃, 3,000 IU; vitamin E, 20 IU; vitamin K₃, 2.5 mg; vitamin B₁, 4 mg; vitamin B₂, 5 mg; vitamin B₃, 30 mg; vitamin B₆, 3 mg; vitamin B₁₂, 0.02 mg; vitamin C, 150 mg; folic acid, 1 mg; D-biotin, 0.1 mg; D-pantothenate, 10 mg; choline chloride (60%), 300 mg; iron (as FeSO₄·H₂O), 65 mg; copper (as CuSO₄·5H₂O), 8 mg; manganese (as MnSO₄·H₂O), 90 mg;zinc (as ZnSO₄·H₂O), 80 mg; iodine (as Ca(IO₃)₂), 0.6 mg; selenium (as Na₂SeO₃), 0.3 mg

**Table S3** The primer sequences of target bacteria in this paper

| **Primer** | **sequence（5’-3’）** | **bp** | **Source** |
| --- | --- | --- | --- |
| *E.coli-F* | GCAAACCACCTTTGGTCG | 195 | [1] |
| *E.coli-R* | CTGTGGGTGTGGATTGACAT |  |  |
| *K.pneumoniae-F* | ACGGCCGAATATGACGAATTC | 68 | [2] |
| *K.pneumoniae-R* | AGAGTGATCTGCTCATGAA |  |  |
| *Kurthia-F* | GGGGAGCAAACAGGATT | 234 | Designed in  this study |
| *Kurthia-R* | TCATTGGGATGTCAAGACC |  |  |
| *Proteus-F* | CAGTGGATTAAGCGCAAATG | 423 | [3] |
| *Proteus-R* | CCTTCAATACGTTCAACAAACC |  |  |

**Table S4** The primer sequences of nitrogen cycling-related genes in this paper

| **Primer** | **sequence（5’-3’）** | **Source** |
| --- | --- | --- |
| *amoA-F* | CTGGGGTTTCTACTGGTGGTC | [4] |
| *amoA -R* | GCAGTGATCATCCAGTTGCG |  |
| *amoB-F* | TGGTAYGACATKAWATGG |  |
| *amoB -R* | RCGSGGCARGAACATSGG |  |
| *narG-F* | TAYGTSGGGCAGGARAAACTG |  |
| *narG -R* | CGTAGAAGAAGCTGGTGCTGTT |  |
| *nirS-F* | TACCACCCSGARCCGCGCGT |  |
| *nirS -R* | GCCGGTGTGVAGGAA |  |
| *nirK-F* | ATCATGGTSCTGCCGCG |  |
| *nirK -R* | GCCTCGATCAGRTTGTGGTT |  |
| *norB-F* | GGNCAYCARGGNTAYGA |  |
| *norB-R* | ACCCANAGRTGNACNACCCACCA |  |
| *nosZ-F* | AGAACGACCAGCTGATCGACA |  |
| *nosZ -R* | TCCATGGTGACGCCGTGGTTG |  |
| *NifH-F* | AAAGGYGGWATCGGYAARTCCACCAC |  |
| *NifH -R* | TGSGCYTTGTCYTCRCGGATBGGCAT |  |


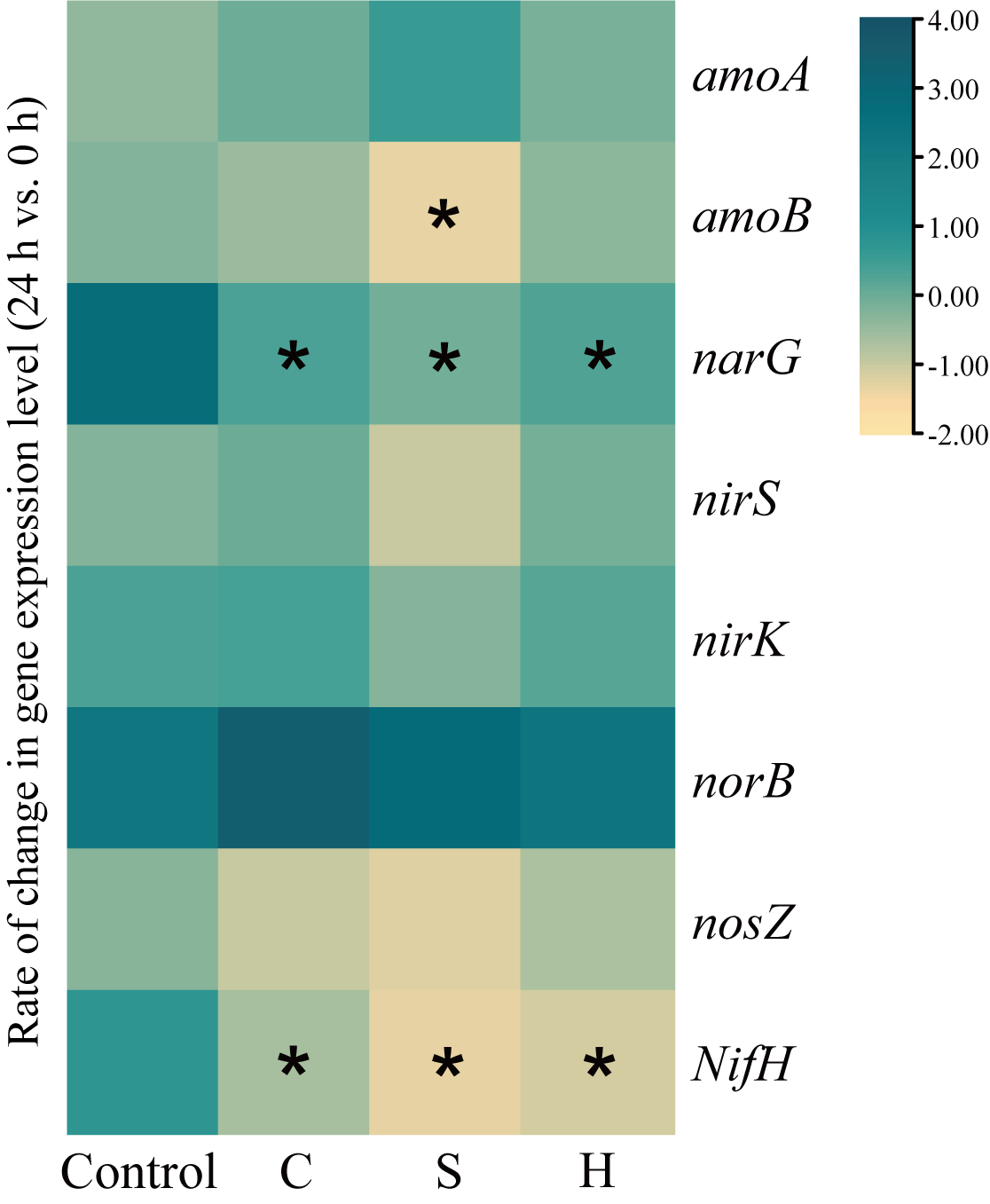


**Fig. S1** Change rate of expression levels of nitrogen cycling gene (24 h vs. 0 h)

**References**

1. Thongsamer T, Neamchan R, Blackburn A, Acharya K, Sutheeworapong S, Tirachulee B, et al. Environmental antimicrobial resistance is associated with faecal pollution in central thailand’s coastal aquaculture region. J Hazard Mater. 2021;416:125718.
2. Dias D, Fonseca C, Mendo S, Caetano T. First characterization of the faecal resistome of eurasian otter (*Lutra lutra*), a sentinel species for aquatic environments. Chemosphere. 2022;309:136644.
3. Mathlum Al-Khafaji KAMA. Original article molecular analysis of virulence genes HpmB and rsbA among *proteus* species isolated from different infectious cases in Iraq. Arch Razi Inst. 2023;78(4):1295–303.
4. Deng W, He J, Chen J, Wu R, Xing S, Liao X. Effects of microplastics on functional genes related to ch4 and n2o metabolism in bacteriophages during manure composting and its planting applications. J Hazard Mater. 2023;460:132288.
